# Autophagy induced by glycosylated CD147 attenuates fibroblast activation and myofibroblast differentiation

**DOI:** 10.1016/j.jbc.2026.113374

**Published:** 2026-07-28

**Authors:** Siqi Long, Shuaichen Ma, Cuncun Chu, Yingying Zhang, Wenyu Zhao, Zhongzheng Li, Ying Cao, Shuang Huang, Juntang Yang, Ivan O. Rosas, Mengshu Cao, Guoying Yu, Lan Wang

**Affiliations:** 1 State Key Laboratory of Cell Differentiation and Regulation, Henan International Joint Laboratory of Pulmonary Fibrosis, Henan Center for Outstanding Overseas Scientists of Organ Fibrosis, Institute of Biomedical Science, College of Life Science, Henan Normal University, Xinxiang, China; 2 Department of Reproductive Medicine, Xinxiang Central Hospital (Fourth Clinical College of Xinxiang Medical University), Xinxiang, China; 3 Division of Pulmonary, Critical Care and Sleep Medicine, Baylor College of Medicine, Houston, Texas, USA; 4 Department of Respiratory and Critical Care Medicine, Nanjing Drum Tower Hospital, Affiliated Hospital of Medical School, Nanjing University, Nanjing, Jiangsu, China

**Keywords:** IPF, CD147, glycosylation, fibroblast, autophagy

## Abstract

Idiopathic pulmonary fibrosis (IPF) is a progressive and invariably fatal interstitial lung disease driven by persistent alveolar epithelial injury, which triggers aberrant fibroblast activation and pathological myofibroblast differentiation. In this study, we identify CD147—a heavily glycosylated transmembrane protein of the immunoglobulin superfamily—as a novel glycosylation-dependent regulator of pulmonary fibrosis operating through dual mechanistic pathways. CD147 expression was markedly downregulated in fibrotic lung tissues and predominantly localized to pulmonary fibroblasts. CD147 depletion significantly enhances fibroblast activation, proliferation, and extracellular matrix (ECM) production. Mechanistically, CD147 promotes autophagosome formation and facilitates p62 degradation, indicating its function in augmenting autophagic clearance of pro-fibrotic effectors. Notably, the anti-fibrotic activity of CD147 is strictly contingent upon its N-linked glycosylation status, the fully glycosylated CD147 robustly suppresses fibroblast-to-myofibroblast transformation and ECM deposition, while non-glycosylated isoforms are entirely devoid of this regulatory capacity. These findings provide compelling evidence that CD147 enhances autophagic clearance of pro-fibrotic effectors and suppresses fibrotic progression through glycosylation-dependent mechanisms.

Idiopathic pulmonary fibrosis (IPF) is a chronic, progressive interstitial lung disease characterized by irreversible scarring of lung parenchyma, leading to progressive respiratory failure (1). As the most common and severe form of idiopathic interstitial pneumonia, IPF predominantly affects individuals aged 50 to 70 years, with a dismal prognosis and median survival of only 3 to 5 years following diagnosis (2). The disease manifests clinically with insidious onset of exertional dyspnea, progressive pulmonary function decline, and characteristic radiological findings, ultimately culminating in terminal respiratory failure (3).

The pathogenic cascade of IPF involves repetitive alveolar epithelial injury that triggers abnormal wound healing responses. Following lung injury, activated epithelial cells secrete profibrotic mediators that induce fibroblast proliferation and differentiation into matrix-producing myofibroblasts (4, 5, 6, 7). This aberrant repair process results in excessive deposition of collagen and other extracellular matrix components, forming the characteristic histopathological features of fibroblastic foci and honeycombing. The resulting architectural distortion and progressive fibrosis severely compromise gas exchange and lung compliance. While the exact etiology remains unknown, current evidence suggests multifactorial pathogenesis involving complex interactions between genetic predisposition (particularly in familial cases), environmental exposures (*e*.*g*., cigarette smoke, occupational dusts), aging-related cellular changes, and metabolic dysregulation (8, 9). Despite advances in understanding these mechanisms, therapeutic options remain extremely limited.

Cluster of differentiation 147 (CD147), a single-chain transmembrane glycoprotein belonging to the immunoglobulin superfamily, is primarily localized on the cell membrane (10, 11). The human CD147 protein, encoded by a 269-amino acid mRNA transcript (12), consists of three distinct structural domains: an extracellular region, a transmembrane domain, and an intracellular region (13). The extracellular N-terminal portion contains two immunoglobulin-like domains (Ig1 and Ig2). Notably, CD147 exhibits variable molecular weights ranging from 31 to 65 kDa on SDS-PAGE, a phenomenon attributed to differential glycosylation at three conserved asparagine (Asn) residues (Asn44, Asn152, and Asn186) (14). These glycosylation sites are strategically positioned within the extracellular region, with one in the Ig1 domain and two in the Ig2 domain. The core protein has a molecular weight of approximately 32 kDa, while post-translational glycosylation increases its mass to 38 to 65 kDa. Importantly, studies have demonstrated that the glycosylation status of CD147 is dynamically regulated through its interactions with various binding partners, including caveolin-1, integrins, monocarboxylate transporters (MCTs), and cyclophilin A (15, 16).

Recent studies have established CD147 as a key mediator in the pathogenesis of various cardiovascular disorders, including ischemic cardiomyopathy (16, 17), heart failure (18), and atherosclerosis (19). The biological activity of CD147 is critically dependent on its post-translational modification, particularly N-glycosylation, which serves as a molecular switch regulating its diverse functions (20). Notably, the glycosylated form of CD147 exhibits distinct functional properties compared to its non-glycosylated counterpart, as exemplified by its essential role in matrix metalloproteinase induction (21). Growing evidence suggests that CD147 glycosylation status significantly influences its involvement in multiple pathological processes, ranging from tumor metastasis to cerebral infarction (22, 23, 24, 25). In the context of cardiac pathophysiology, glycosylated CD147 has been shown to drive pathological myocardial hypertrophy through a specific molecular mechanism involving TRAF2 binding and subsequent activation of the TRAF2-TAK1 signaling pathway, ultimately leading to adverse cardiac remodeling and dysfunction (26). Despite these important advances in understanding CD147 glycosylation in cardiovascular and other diseases, its potential role in pulmonary fibrosis remains completely unexplored. This significant knowledge gap motivates our current investigation into the contribution of CD147 and its glycosylation modifications to the pathogenesis of pulmonary fibrosis.

This study aimed to elucidate the functional relationship between CD147 glycosylation and pulmonary fibrosis progression. Our findings indicate that upregulated CD147 expression suppresses fibroblast activation and subsequent transformation into collagen-producing myofibroblasts, while concurrently enhancing autophagic flux in fibroblasts to promote extracellular matrix degradation. Importantly, we identified that these anti-fibrotic activities are strictly dependent on CD147's glycosylation status, establishing a novel post-translational regulatory mechanism in fibrosis development. The differential effects mediated by glycosylated *versus* non-glycosylated CD147 isoforms suggest that selective modulation of this post-translational modification could represent an innovative strategy for developing targeted anti-fibrotic therapies to mitigate disease progression.

## Results

### CD147 is downregulated in fibrotic lung tissues and predominantly expressed in fibroblasts

**Figure 1:**
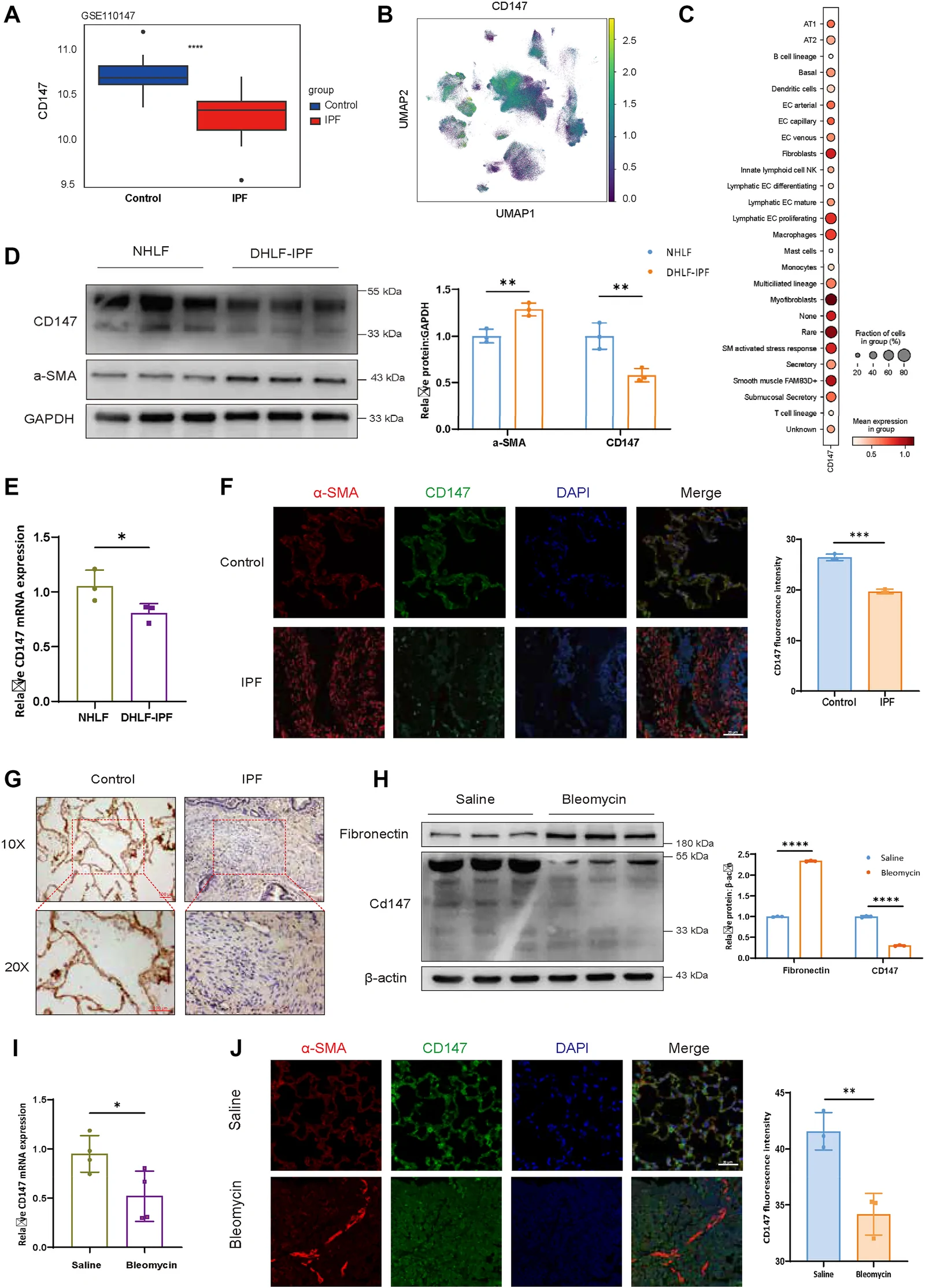
**CD147 was downregulated in fibrotic lung tissues.***A*, the expression levels of CD147 were obtained from the GEO database (GSE110147) in patient lung tissues of IPF and normal lung tissue. *B*, the expression of CD147 in lungs as predicted for single cell types. Circles mark Fibroblasts cells. *C*, the dotplot depicts a gradient of marker gene expression across the activated cell types. CD147 showed Expressed mainly on human Fibroblasts cells. *D*, Western blot showing the levels of CD147 and a-SMA in primary human lung fibroblasts from IPF patients and healthy controls. *E*, mRNA levels of CD147 in human lung fibroblasts from IPF patients and healthy controls. *F*, representative immunofluorescence using anti–a-SMA (Fibroblasts marker, *red*) and anti-CD147 (*green*) antibodies, showing reduction in CD147 expression in lung fibroblasts from honeycombs in IPF lung compared with healthy controls. *G*, immunohistochemical staining of CD147 in lung fibroblasts from honeycombs in IPF lung compared with healthy controls. *H*, representative results of CD147 expression of protein level and quantitative analysis in mice lung tissues after BLM induction for 21 days (*I*). The transcriptional change in CD147 expression was analyzed using qRT-PCR in mice induced with saline or bleomycin. *J*, representative images of paraffin sections of lung tissue from a mouse model of BLM-induced pulmonary fibrosis, CD147 (*green*), α-SMA (*red*), DAPI (*blue*), Scale bars, 20 μm. *∗p* < 0.05, *∗∗∗∗p* < 0.0001.

To investigate the role of CD147 in pulmonary fibrosis, we evaluated its expression in both IPF patients and a bleomycin-induced murine fibrosis model (Fig. S1, *A* and *B*). Analysis of the GEO dataset (GSE110147) demonstrated a significant reduction in CD147 expression in the lung tissues of IPF patients compared to controls (Fig. 1*A*). Further examination of single-cell RNA-seq data from the Human Lung Cell Atlas revealed that CD147 was predominantly expressed in fibroblasts and myofibroblasts, as illustrated in the dot plot and UMAP (Fig. 1, *B* and *C*). A significant decrease CD147 expression of mRNA or protein levels was able to verify in diseased human lung fibroblasts from patients with idiopathic pulmonary fibrosis (DHLF-IPF) when compared with normal human lung fibroblasts (NHLF) (Fig. 1, *D* and *E*). Meanwhile, low protein levels of CD147 in IPF fibroblasts were supported by immunofluorescence (IF) and immunohistochemistry (IHC) (Figs. 1, *F* and *G*). In addition, bleomycin-treated mice exhibited a marked decrease in CD147 expression at both the protein (Figs. 1*H* and S1*D*) and mRNA (Fig. 1*I*) levels compared to saline-treated controls. IHC analysis further localized CD147 expression primarily to fibroblasts in healthy lung tissue, whereas its expression was significantly diminished in fibrotic lesions of bleomycin-induced lungs (Fig. S1*C*). Co-staining with the fibroblast marker α-smooth muscle actin (α-SMA) confirmed that CD147 expression was particularly reduced in regions with severe fibrotic pathology (Fig. 1*J*). Collectively, these findings demonstrate that CD147 is downregulated in fibrotic lung tissues and is predominantly associated with fibroblasts, suggesting its potential involvement in the pathogenesis of pulmonary fibrosis.

### CD147 depletion promotes fibroblast activation and profibrotic responses

**Figure 2:**
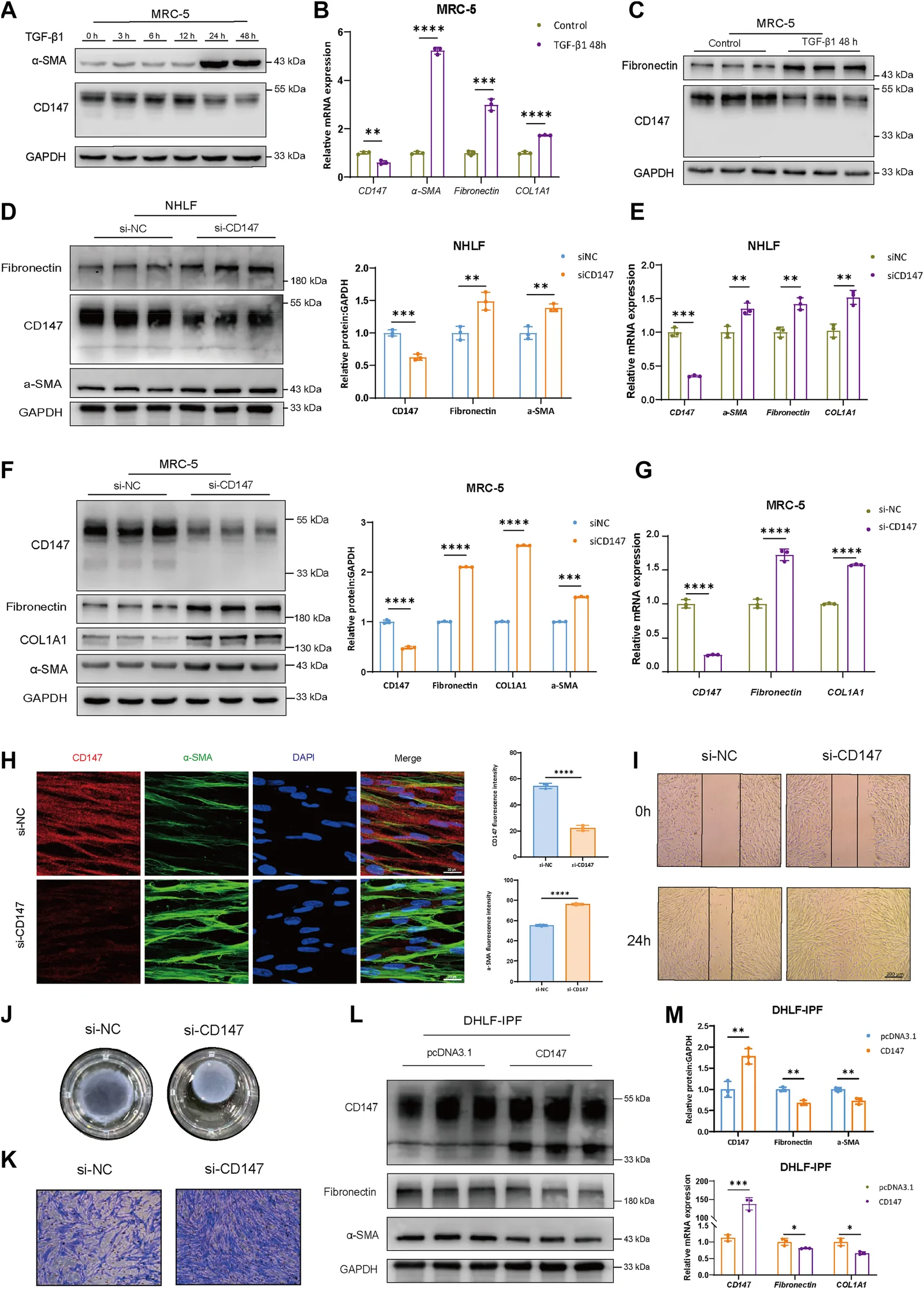
**CD147 depletion facilitates the activation of fibroblasts.***A*, Western blot was used to detect the protein expression in MRC-5 cells after treatment with TGF-β1 (5 ng/ml) in a time-graded manner. *B*, RT-qPCR was utilized to investigate the gene changes in MRC-5 cells after 48 h of treatment with TGF-β1. (*C*) Western blot was performed to examine the protein expression in MRC-5 cells after 48 h of treatment with TGF-β1 (5 ng/ml) and the corresponding quantitative analysis. *D*, WB analysis of the protein expression of Fibronectin, CD147, and a-SMA in HLF cells after knockdown of CD147. *E*, quantitative RT–PCR analysis of relative, *CD147*, *Fibronectin*, *COL1A1*, and *a*-*SMA* mRNA expression in HLF cells after knockdown of CD147. *F*, Western blot was used to detect the protein expression and perform quantitative analysis in MRC-5 cells after knocking down CD147. *G*, RT-qPCR was employed to investigate the mRNA changes in MRC-5 cells after knocking down CD147. *H*, immunofluorescence staining images of CD147 (*red*), α-SMA (*green*), and DAPI (*blue*) in MRC-5 cells after knocking down CD147 expression, with scale bars of 20 μm. *I*, cell scratch assay was carried out to evaluate the representative image and quantitative analysis of cell migration ability in MRC-5 cells after knocking down CD147 expression, with scale bars of 100 μm. *J*, the collagen contraction assay were performed to evaluate the activation capacities of MRC-5 cells after knocking down CD147 expression. *K*, Transwell assay was performed to assess the representative image and quantitative analysis of cell migration ability in MRC-5 cells after knocking down CD147 expression, with scale bars of 100 μm. *L*, WB analysis of the protein expression of Fibronectin, CD147 and a-SMA after overexpression of CD147 in DHLF-IPF cells. *M*, quantitative RT–PCR analysis of the relative mRNA expression levels of CD147, Fibronectin and COL1A1 in DHLF-IPF cells after overexpressing CD147. *∗p* < 0.05, *∗∗p* < 0.01, *∗∗∗p* < 0.001, *∗∗∗∗p* < 0.0001.

To explore the functional role of CD147 in the pathogenesis of pulmonary fibrosis, we first examined its expression dynamics in TGF-β1–stimulated human embryonic lung fibroblasts (MRC-5 cells). Time-course experiments revealed a progressive decrease in CD147 protein levels, with maximal downregulation observed at 48 h post-TGF-β1 treatment (Fig. 2*A*). Consistent with this, prolonged TGF-β1 exposure (48 h) significantly suppressed CD147 expression at both mRNA and protein levels, concomitant with upregulation of key fibrotic markers (COL1A1, Fibronectin, and α-SMA) (Fig. 2, *B* and *C*). To determine whether CD147 loss directly contributes to fibroblast activation, we performed siRNA-mediated knockdown in isolated primary NHLF. CD147 depletion significantly down-regulated the level of α-SMA and Fibronectin in human lung fibroblasts (Fig. 2, *D* and *E*). Similar results were obtained for MRC-5 human lung fibroblasts; CD147-deficient MRC-5 exhibited pronounced upregulation of myofibroblast markers (Fibronectin, COL1A1 and α-SMA) at both transcriptional and translational levels (Figs. 2, *F* and *G*, S2*A*). Immunofluorescence staining for α-SMA further validated these findings, demonstrating significantly increased fluorescence intensity in CD147-silenced cells (Fig. 2*H*). Notably, CD147 silencing also augmented collagen gel contraction—a hallmark of fibroblast activation—suggesting enhanced contractile and ECM-remodeling capabilities (Fig. 2*J*). Consistently, CD147 depletion markedly enhanced fibroblast viability, proliferation, migration, and clonogenic capacity (Figs. 2, *I* and *K* and S2, *B* and *C*). Conversely, CD147 overexpression in DHLF and MRC5 cells led to significantly increased protein and mRNA expression levels of the fibrotic markers fibronectin and α-SMA (Figs. 2, *L* and *M* and S2*D*).

Taken together, these data demonstrate that CD147 depletion promotes fibroblast activation, proliferation, and ECM synthesis, suggesting its critical role in restraining fibrogenic responses.

### CD147 activates autophagy and accelerates P62 degradation in lung fibroblasts

**Figure 3:**
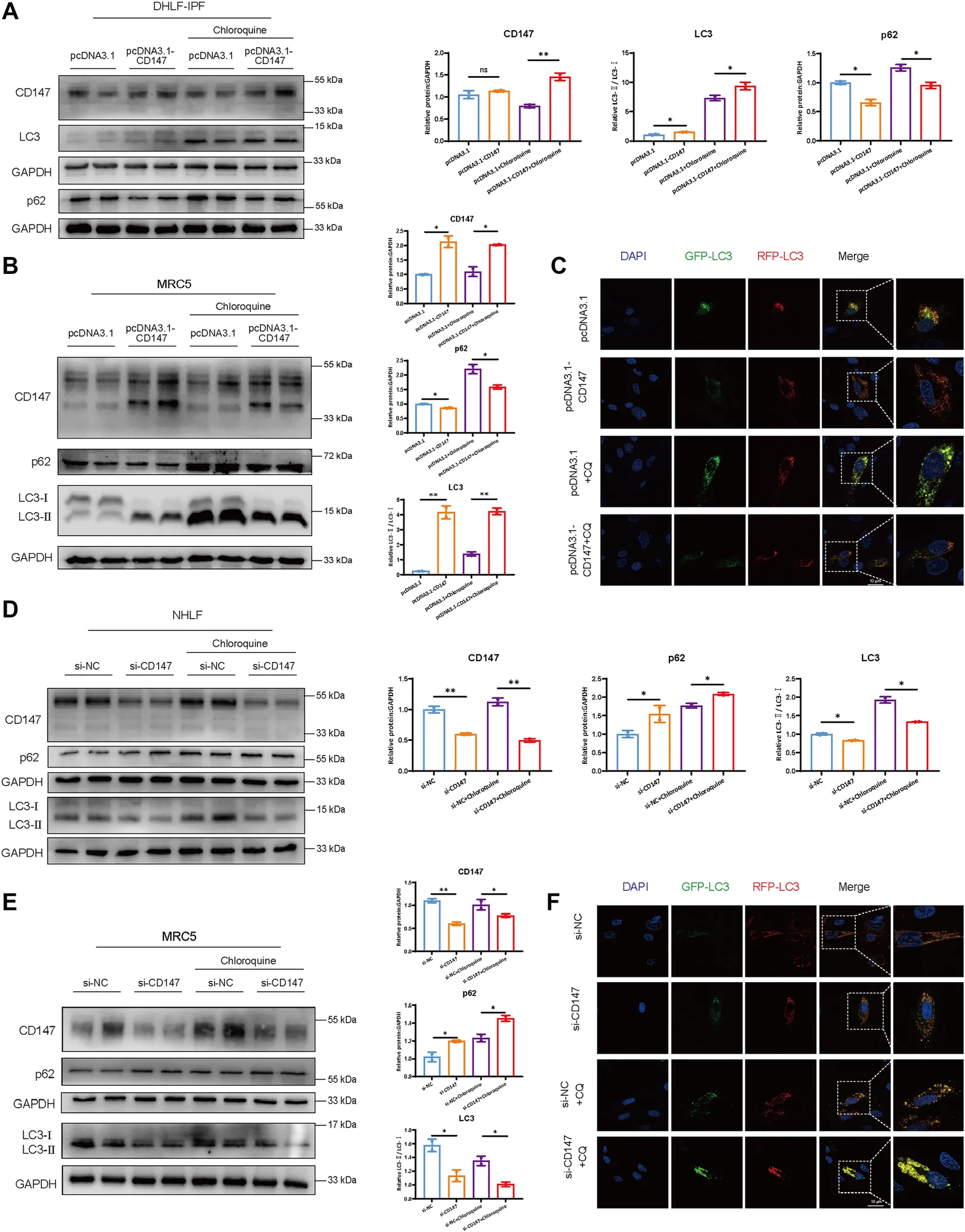
**CD147 promotes autophagy and leads to P62 degradation in lung fibroblasts.***A*, Western blot was used to detect the impact of overexpressing CD147 on the expression of autophagy-related proteins in DHLF-IPF cells. *B*, Western blot was used to detect the impact of overexpressing CD147 on the expression of autophagy-related proteins in MRC-5 cells, along with quantitative analysis. *C*, immunofluorescence staining of GFP/RFP-LC3 in MRC-5 cells treated with CQ (50 μm) for 4 h after pre-treatment with Overexpression of CD147. *D*, Western blot was used to detect the impact of knocking down CD147 on the expression of autophagy-related proteins in MRC-5 cells, along with quantitative analysis. *E*, Western blot was used to detect the impact of knocking down CD147 on the expression of autophagy-related proteins in in HLF cells. *F*, immunofluorescence staining of GFP/RFP-LC3 in MRC-5 cells treated with (CQ, 50 μm) for 4 h after pre-treatment with knocking down CD147. *∗p* < 0.05, *∗∗p* < 0.01, *∗∗∗p* < 0.001, *∗∗∗∗p* < 0.0001.

Autophagy serves as a crucial mechanism for the clearance of excess cytoplasmic collagen, contributing to the maintenance of ECM homeostasis and cellular proteostasis. To investigate whether CD147 modulates this process, we evaluated its impact on autophagic activity in fibroblasts. Chloroquine inhibits autophagy by alkalinizing lysosomal pH, which impairs lysosomal function and blocks autophagosome-lysosome fusion, ultimately suppressing protein degradation. Strikingly, CD147 overexpression in DHLF and MRC5 cells treated with chloroquine resulted in decreased p62 and elevated LC3-II levels, reflecting increased autophagosome accumulation (Fig. 3, *A* and *B*). To further confirm the involvement of CD147 in autophagy, MRC-5 cells co-transfected with a GFP-RFP-LC3 reporter and either CD147 or control plasmid were treated with Chloroquine. Overexpression of CD147 accelerated green fluorescence quenching and significantly reduced red/green fluorescence colocalization compared to the vector control group (Fig. 3*C*). Conversely, CD147 knockdown in MRC-5 cells led to a marked increase in p62 protein levels (Fig. 3, *D* and *E*). This was accompanied by attenuated green fluorescence quenching and increased red/green colocalization relative to the negative control group, indicating that CD147 depletion robustly promotes autophagic flux (Fig. 3*F*). Collectively, these results demonstrate that CD147 facilitates autophagosome formation and accelerates p62 degradation, underscoring its role in promoting autophagic clearance.

### Glycosylated CD147 suppresses fibroblast-to-myofibroblast differentiation through N-linked glycosylation-dependent mechanisms

**Figure 4:**
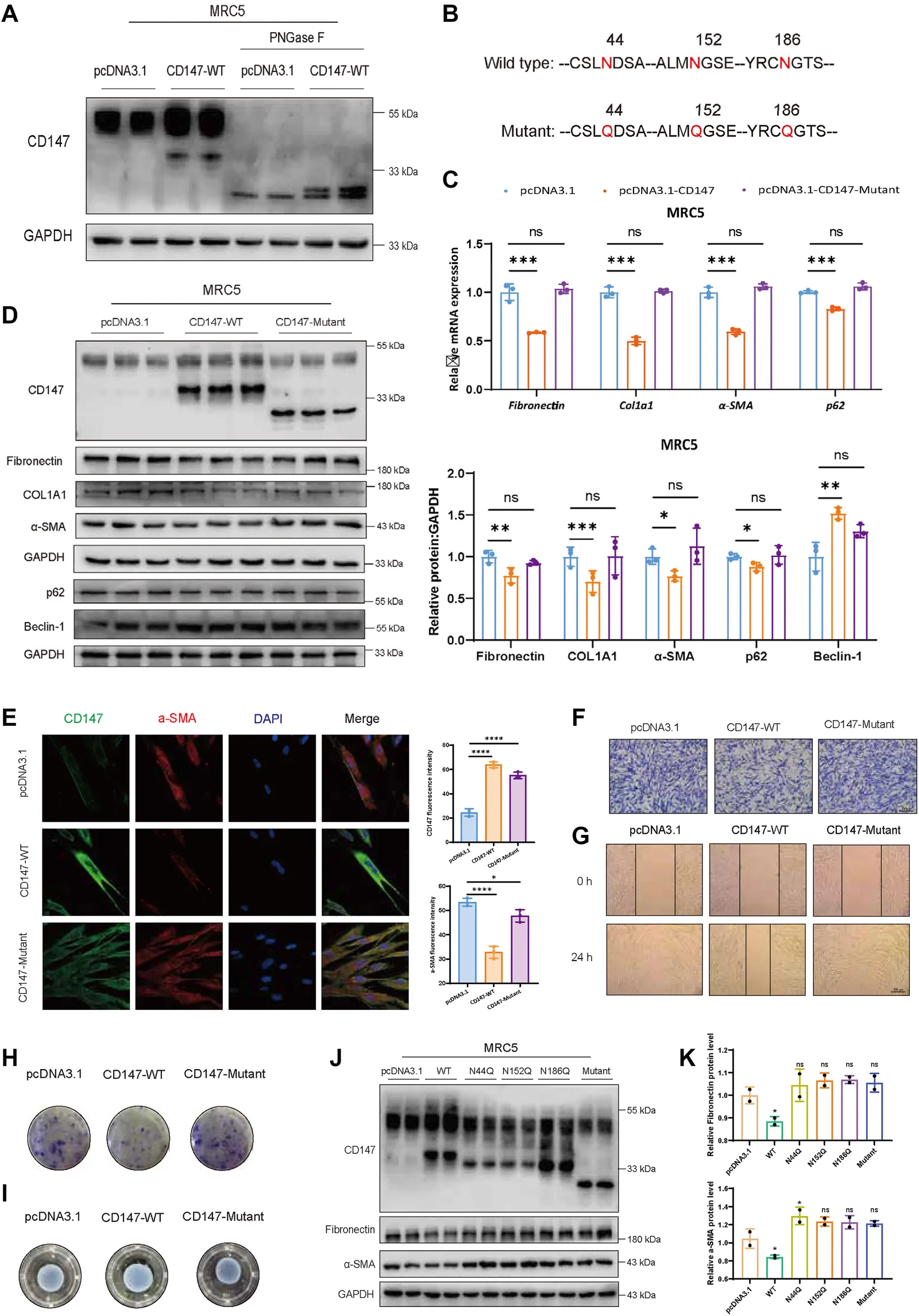
**Glycosylated CD147 Suppresses Fibroblast-to-Myofibroblast Differentiation.***A*, Western blot was performed to detect the changes in CD147 protein molecular weight expression after treating MRC-5 cell protein samples with PNGase F. *B*, Mutagenesis of CD147 glycosylation sites. Three conserved asparagine (Asn) glycosylation sites (N44, N152, and N186) in human CD147 were converted to glutamine (Gln) to generate glycosylation-defective CD147 protein. *C*, RT-qPCR was employed to examine the mRNA changes in MRC-5 cells overexpressing wild-type CD147 and glycosylation site mutant CD147. *D*, Western blot was used to detect the protein expression in MRC-5 cells overexpressing wild-type CD147 and glycosylation site mutant CD147, with quantitative analysis graphs. *E*, immunofluorescence of CD147 (*green*), α-SMA (*red*), and DAPI (*blue*) in MRC-5 cells overexpressing wild-type CD147 and glycosylation site mutant CD147, with scale bars of 20 μm. *F*, Transwell assay was conducted to assess the cell migration ability in MRC-5 cells overexpressing wild-type CD147 and glycosylation site mutant CD147, with representative and quantitative analysis graphs, scale bars of 100 μm. *G*, scratch assay was performed to evaluate the cell migration ability in MRC-5 cells overexpressing wild-type CD147 and glycosylation site mutant CD147, with representative and quantitative analysis graphs, scale bars of 100 μm ns: not significant. *H*, CFU assay was used to evaluate the colony-forming ability in MRC-5 cells overexpressing wild-type CD147 and glycosylation site mutant CD147, with representative and quantitative analysis graphs. *I*, the collagen contraction assay were performed to evaluate the activation capacities of MRC-5 cells after overexpressing wild-type CD147 and glycosylation site mutant CD147. (*J*) HA-tagged WT, N44Q, N152Q, N186Q and N44/152/186Q CD147 were transiently transfected into MRC-5 cells. Cell extracts were immunoblotted with anti-HA, anti-CD147, anti-α-SMA or anti-Fibronectin antibody. *K*, Quantitative data derived from (*J*).. ns: not significant, *∗p* < 0.05 *∗∗p* < 0.01, *∗∗∗p* < 0.001.

The previous analyses revealed that CD147 predominantly exists in its glycosylated form in both murine lung tissue and fibroblasts, suggesting that glycosylation plays a crucial role in its regulatory functions. To characterize the glycosylation status of CD147, we employed PNGase F, an enzyme that cleaves N-glycans between the innermost N-acetylglucosamine (GlcNAc) and asparagine residues, effectively removing high-mannose, hybrid, and complex oligosaccharides. Western blot analysis of PNGase F-treated fibroblast lysates demonstrated that CD147 primarily exists in glycosylated forms (38-65 kDa), as evidenced by the disappearance of these bands and concomitant increase in the intensity of the non-glycosylated 32 kDa band (Fig. 4*A*). This finding, coupled with the conservation of all three predicted N-glycosylation sites, strongly suggests that CD147 is heavily glycosylated in fibroblasts.

To investigate the functional significance of CD147 glycosylation, we performed site-directed mutagenesis, substituting asparagine with glutamine at key glycosylation sites either individually or in combination (Fig. 4*B*). Strikingly, overexpression of wild-type (WT) CD147 significantly downregulated both protein and mRNA expression of fibrotic markers (Fibronectin, COL1A1, and α-SMA) and p62, while glycosylation-deficient mutants failed to affect these markers (Fig. 4, *C* and *D*). Immunofluorescence analysis further confirmed the reduced α-SMA fluorescence intensity in WT-CD147-overexpressing fibroblasts but not in cells expressing glycosylation mutants (Fig. 4*E*). Notably, we observed distinct colocalization patterns of α-SMA in mutant-expressing cells, suggesting impaired regulatory function. Functional assays revealed that WT-CD147, but not glycosylation mutants, significantly inhibited fibroblast migration capacity (Fig. 4, *F* and *G*), colony formation (Fig. 4*H*), and collagen gel contraction (Fig. 4*I*). Intriguingly, single-point mutations at N44, N152, or N186 all resulted in an approximately 35 kDa band shift but failed to recapitulate WT-CD147's ability to suppress fibrotic marker expression (Fig. 4*J*), indicating that complete glycosylation is essential for CD147's antifibrotic activity. These findings demonstrate that CD147's ability to suppress fibroblast activation and ECM production is strictly dependent on its N-linked glycosylation status. While fully glycosylated CD147 effectively inhibits fibroblast-to-myofibroblast differentiation and associated fibrotic processes, non-glycosylated variants lose this regulatory capacity.

### Glycosylated CD147 attenuates bleomycin-induced pulmonary fibrosis *in vivo*

**Figure 5:**
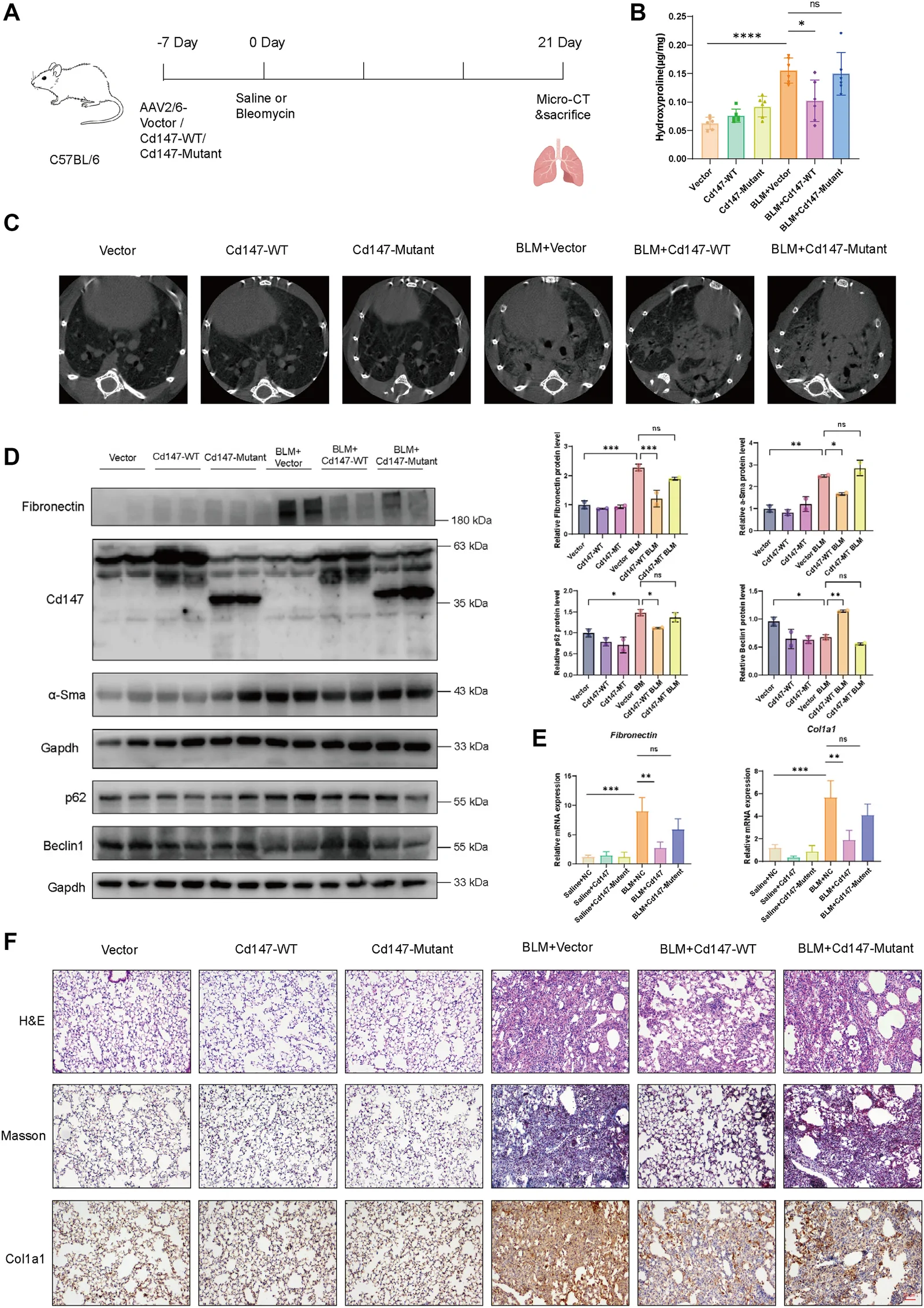
**Glycosylated CD147 Inhibits bleomycin-induced mouse lung fibrosis.***A*, the schematic diagram depicts the timeline for intratracheal instillation of AAV2/6, AAV2/6-CD147-WT, AAV2/6-CD147-Mutant, and bleomycin in a mouse model of bleomycin-induced pulmonary fibrosis. *B*, hydroxyproline content analysis was performed on the entire right lung. *C*, representative axial micro-CT images of the mouse lungs following 21 days of exposure to bleomycin. *D*, Western blot was used to detect the protein expression of wild-type CD147 and glycosylation site mutant CD147, with quantitative analysis graphs. *E*, qRT-PCR analysis the changes in gene expression of *fibronectin* and *col1a1* in lung tissues. *F*, representative photomicrographs of H&E, Masson, and COL1A1 IHC on lung sections from each group of mice. *ns*, not significant, *∗p* < 0.05 *∗∗p* < 0.01, *∗∗∗p* < 0.001.

To investigate the anti-fibrotic effects of CD147 glycosylation *in vivo*, we employed a well-established bleomycin-induced pulmonary fibrosis model in C57/BL6N mice. Seven days prior to bleomycin challenge (1.5 U/kg, intratracheal), mice were administered AAV2/6 vectors expressing either CD147-WT or CD147-Mutant *via* the same route (Fig. 5*A*). Comprehensive analyses revealed that glycosylated CD147 exerted significant protective effects against pulmonary fibrosis. Hydroxyproline content quantification demonstrated that CD147-WT, but not the glycosylation mutant, markedly reduced bleomycin-induced collagen deposition (Fig. 5*B*). Micro-CT imaging before sacrifice showed that CD147-WT overexpression preserved lung architecture, as evidenced by reduced dense infiltrates and improved lung opacity compared to bleomycin-only controls, while the glycosylation mutant failed to confer such protection (Fig. 5*C*). Western blot, qRT-PCR and immunohistochemical analyses consistently showed that CD147-WT significantly downregulated expression of Fibronectin and Col1a1 in bleomycin-treated lungs, and upregulated expression of autophagy-related proteins including Beclin-1and p62 (Fig. 5, *D* and *E*). Histopathological evaluation through H&E and Masson's trichrome staining further confirmed that CD147-WT effectively attenuated fibrotic lesion formation and collagen accumulation (Fig. 5*E*). Collectively, these findings provide compelling *in vivo* evidence that the anti-fibrotic activity of CD147 is strictly dependent on its glycosylation status, highlighting glycosylated CD147 as a potential therapeutic target for pulmonary fibrosis intervention.

## Discussion

While CD147 has been well-characterized in various malignancies, its role in IPF and the functional significance of its glycosylation remain largely unexplored. Our study provides the first evidence that CD147 exerts glycosylation-dependent anti-fibrotic effects in pulmonary fibrosis, offering new insights into IPF pathogenesis and potential therapeutic strategies.

The pathogenesis of IPF, though incompletely understood, is believed to originate from dysregulated wound-healing responses following repetitive alveolar injury (27, 28). This process involves a vicious cycle of epithelial injury, aberrant repair, and progressive fibrosis. Injured alveolar epithelial cells release pro-fibrotic mediators that drive persistent fibroblast activation and myofibroblast differentiation (29), disrupting the critical balance between epithelial regeneration and fibrotic responses (5, 30, 31). The resulting excessive ECM deposition remodels lung architecture, impairing gas exchange and lung mechanics, ultimately leading to respiratory failure (32, 33). Fibroblasts serve as central orchestrators of this process. Following chronic injury, epithelial-derived signals, including surfactant proteins, promote fibroblast-to-myofibroblast transition (4, 7). These activated myofibroblasts become the primary ECM-producing cells, creating a self-perpetuating fibrotic microenvironment that progressively replaces functional alveoli with scar tissue (34).

Our understanding of IPF pathophysiology must also consider the role of autophagy, a critical cellular quality control mechanism. While autophagy serves protective functions during acute injury by clearing cellular debris (35, 36), IPF patients exhibit characteristic autophagic dysfunction—manifested by impaired autophagosome–lysosome fusion, accumulated p62/ubiquitinated proteins, and overall reduced autophagic flux (37). This paradoxical suppression of autophagy during chronic inflammation appears to contribute to fibrotic progression by preventing the clearance of pathogenic proteins and ECM components (38), highlighting the complex, context-dependent role of autophagy in fibrosis.

CD147, a ubiquitously expressed glycoprotein, displays tissue-specific glycosylation patterns that are modulated through interactions with caveolin-1, monocarboxylate transporters (MCTs), and cyclophilins (20). Our findings demonstrate that glycosylated CD147 expression is markedly diminished in BLM-induced pulmonary fibrosis, while genetic knockdown of CD147 potentiates fibroblast activation, proliferation, migratory capacity, and ECM production. Importantly, we establish that only the glycosylated form of CD147 - and not its non-glycosylated counterpart - exerts suppressive effects on fibroblast activation and ECM deposition. This anti-fibrotic activity is strictly contingent upon CD147's glycosylation status, as evidenced by the functional disparity between wild-type and glycosylation-deficient mutants. These collective findings identify glycosylated CD147 as both a critical regulator of fibroblast activation and a promising molecular target for pulmonary fibrosis intervention, with its glycosylation-dependent activity suggesting the existence of precise, targetable mechanisms that could be therapeutically exploited.

While our study establishes CD147 glycosylation as a critical regulator of fibroblast behavior in pulmonary fibrosis, several key questions warrant further investigation to provide a more comprehensive understanding of CD147's multifaceted role in fibrosis pathogenesis but may also pave the way for developing novel glycosylation-targeted therapeutic strategies for IPF and other fibrotic disorders.

## Experimental procedures

### Clinical samples

IPF lung tissues and control non-IPF lung tissue samples were obtained following the American Thoracic Society/European Respiratory Society/Japanese Respiratory Society/Latin American Thoracic Association clinical practice guidelines33 at Henan Provincial Chest Hospital. IPF lung tissue samples were obtained from the remains of a patient’s surgical biopsy or lung transplant. The control groups were taken from normal lesion-free edge tissue of the lung cancer specimen. The research protocol for human lung tissue samples received approval from the Henan Provincial Chest Hospital Medical Research Ethics Committee (number 2019-05-07), and informed consent was diligently obtained from all patients before any surgical procedures. The study strictly adhered to the principles outlined in The Code of Ethics of the World Medical Association (Declaration of Helsinki) governing experiments involving human subjects.

### MRC-5 cell culture

MRC-5 cells were purchased from ATCC and cultured in MEM/EBSS medium supplemented with 10% fetal bovine serum and 1% Streptomycin and penicillin, which were placed in a constant temperature incubator at 37 °C with 5% carbon dioxide. Trypsin was used for digesting cells that were 90% healed, for subsequent experiments.

### Isolation of primary lung fibroblasts

Primary lung fibroblasts were isolated following established procedures. Initially, lung tissues were dissected into small cubes and subjected to collagenase (1 mg/ml; C0130; Sigma) digestion in Hanks’ balanced salt solution (Hyclone) for 30 min at 37 C, followed by centrifugation at 500*g* for 5 min. The tissues were subsequently washed and resuspended in low-glucose Dulbecco’s modified Eagle’s medium (Hyclone) containing 15% fetal bovine serum, 100 U/ml penicillin, and 100 mg/L streptomycin (Solarbio). After allowing the tissues to adhere overnight at 37 C with 5% CO_2_, the culture medium was gently replaced to eliminate any nonadherent cells and debris, and cultured for an additional 5 days before the initial passage. Confluent cells within four passages were then used in subsequent experiments.

### Mouse bleomycin-induced lung fibrosis model

Male wild-type C57BL/6 (8-10 weeks of age) mice were purchased from the Charles River (Beijing, China). The mice were kept in a special room with constant temperature and humidity and adequate water and food. Animal husbandry and experimental procedures were performed following the guidelines of the Animal Care and Use Committee of Henan Normal University (IACUC, SMKX-2118BS1018), which in accordance with the guidelines of Animal Behavior Associations and national regulations. Mice were anesthetized with isoflurane and suspended on an operating table. Mice were intratracheal instilled with 50 μl of AAV2/6-Voctor 、AAV2/6-CD147-WT or AAV2/6-CD147-Mutant virus. The AAV were synthesized by Hanbio Biotechnology. One week later, the mice were challenged of BLM (1.5 U/kg) dissolving in saline or equal volume of saline. Each group consisted of 10 mice. The body weight of the mice was measured every other day during the experiment. After challenged of BLM for 21 days, the mice were anesthetized and the lung tissue samples were collected.

### Construction of overexpression plasmid

Overexpression of CD147 was achieved by targeting the sequences 5′- CGCGGATCCGCCACCATGGCGGCTGCGCTGTTCGTGCTGCTGGGATTCGCGCTGCTGGG-3′ (forward), 5′-CCGCTCGAGTCAAGCGTAGTCTGGGACGTCGTATGGGTAGGAAGAGTTCCTCTGGCGGAC-3′ (reverse) in the pcDNA3.1-Vector. Glycosylation site mutant of CD147 overexpression plasmid was purchased from Generay Biotechnology.

### Overexpressing and silencing of CD147

HA-tagged CD147 were cloned into pcDNA3.1 vector by a standard subcloning procedure. The CD147 siRNA and a negative control were synthesized by RiboBio. Cells were transfected with the plasmid or siRNA following to the manufacturer's instruction. After 24 or 48 h, the transfected cells were used for follow-up experiments.

### Transwell and cell scratch test

Cells were resuspended in serum-free MEM/EBSS medium and inoculated into the upper cavity coated with a transwell insert without BD matrix glue, and the lower cavity was filled with MEM/EBSS containing FBS (20%). By counting the number of cells in five random fields, the migrated cells were stained, imaged, and analyzed. Cells were plated and grown into confluent monolayers in six-well plates. Then, the pipette tip was used to create scratches. After the injury, the process of cell migration was observed by microscope at 0 h、24 h and 48 h.

### Deglycosylation with PNGase F

Digestion was performed as described by the manufacturer. Briefly, purified native CD147 was denatured in the supplied buffer and was digested with PNGase F for 1 h and 4 h at 37 °C in 1 × G7 buffer before resolving by SDS/PAGE. In the oligomerization studies, the denaturation procedure was omitted and the protein was digested under native conditions for 24 h.

### Immunofluorescent staining

Cells were fixed with 4% paraformaldehyde for 10 min, then neutralized with 2 mg/ml glycine for excess aldehyde groups, permeabilized with 1.5‰ TritonX-100 for 20 min, and blocked with 10% goat serum for 30 min. Cells were incubated with CD147, α-SMA, Col1a1, Ki-67 and HA-tag were visualized with an overnight with specific fluorochrome primary antibodies including CD147 (Proteintech, 11989-1-AP), α-SMA (Proteintech, 14395-1-AP), COL1A1 (Proteintech, 86093-1-AP), Ki-67 (CST, 9449) and HA-tag (CST, 3724) at a concentration of 1:100. After extensive washing with PBS, cells were incubated with goat Alexa Fluor 488-labeled secondary antibody (CST, 89853) or Alexa Fluor 594-labeled secondary antibody (CST, 8889) for 1 h at room temperature and nuclei were stained with DAPI for 10 min. The images were obtained by using a confocal laser scanning microscope (Germany).

### Western blot

Cells were lysed with SDS, and the cells were made non-viscous using sonication. Additionally, lung tissues were lysed in the RIPA lysis buffer (P0013B, Beyotime) supplied with protease and phosphatase inhibitors. Protein concentration was quantified using a BCA protein assay Kit (Solarbio). Protein samples of the same mass were run by SDS-PAGE and then transferred to PVDF membranes, blocked with 5% skim milk for 40 min, and incubated with primary antibodies overnight. After being washed with TBST three times, membranes were then incubated with IRDye 800CW- or 680RD-conjugated secondary antibodies and visualized using a LI-COR Odyssey Imaging System (LI-COR Biosciences, Lincoln, NE, United States).

### Quantitative real-time PCR

Cells were extracted with Trizol reagent 48 h after transfection for RNA extraction. cDNA was synthesized using a reverse transcription kit (Promega Corporation). Subsequently, according to the manufacturer’s instructions, qRT-PCR was performed using a SYBR green kit (Yeasen) under a Light Cycler 96 fluorescent quantitative PCR system (Roche). ACTB was used as an internal reference, and the data were calculated using the 2−ΔΔCt method. The expression of Col1a1, Fibronectin, CD147 and α-SMA. Primers for RT-qPCR are listed in Table 1.

**Table 1:** List of primers used in this study

Primer name	Sequence (5′-3′)
H-CD147-F	GGCTGTGAAGTCGTCAGAACAC
H-CD147-R	ACCTGCTCTCGGAGCCGTTCA
M-CD147-F	AGAGGACACAGGCACTTACGAG
M-CD147-R	GACTTCCTGGACAGAGGTTTGG
H-Col1a1-F	GAGGGCCAAGACGAAGACATC
H-Col1a1-R	CAGATCACGTCATCGCACAAC
H-Fibronectin-F	ACAACACCGAGGTGACTGAGAC
H-Fibronectin-R	GGACACAACGATGCTTCCTGAG
H-α-SMA-F	CTCTGGACGCACAACTGGCATC
H-α-SMA-R	CACGCTCAGCAGTAGTAACGAAGG
H-GAPDH-F	ACCTCCAACCCCGAGAACA
H-GAPDH-R	ACCACCCTGTTGCTGTAGCCAA
H-ACTB-F	CACCATTGGCAATGAGCGGTTC
H-ACTB-R	AGGTCTTTGCGGATGTCCACGT
M-ACTB-F	CATTGCTGACAGGATGCAGAAGG
M-ACTB-R	TGCTGGAAGGTGGACAGTGAGG

### CCK-8 assay procedure

Cells were seeded in a 96-well plate at a density of 1000 cells/well and cultured for 24 h. After treatment with experimental compounds (or vehicle control), 10 μl of CCK-8 reagent was added to each well, followed by incubation for 1 to 4 h at 37 °C. Absorbance was measured at 450 nm using a microplate reader, with blank wells (medium + CCK-8) used for background subtraction. Cell viability was calculated as:

(ODtreated−ODblank)/(ODcontrol−ODblank)×100%(ODtreated−ODblank)/(ODcontrol−ODblank)×100%

Data represent mean ± SD of triplicate experiments.

### Immunohistochemistry

The expressions of α-SMA, Fibronectin, CD147 and COL1A1 were characterized by immunohistochemistry using specific antibodies. Lung tissue slices were deparaffinized and rehydrated in xylene, permeabilized with 3‰ Triton, blocked with 3% endogenous peroxidase, subjected to antigen heat repair in sodium citrate buffer (pH 6.0), incubated overnight with antibodies against Fibronectin (Proteintech, 1:300), α-SMA (Proteintech, 14395-1-AP), CD147 (Proteintech, 11989-1-AP) and COL1A1 (Proteintech, 86093-1-AP). After extensive washing with PBS, lung tissue slices were incubated with secondary antibody and SABC regent (Beyotime, P0101) for 1 h at room temperature and nuclei were stained with Hematoxylin reagent and visualized with DAB. Image was obtained using an upright microscope (Nikon).

### Masson trichrome stain

Lung tissues were deparaffinized and rehydrated in xylene, nucleated with hematoxylin, differentiated with 1% ethanol hydrochloric acid, stained with Poncein, immersed in 2% glacial acetic acid for a moment, treated with 1% phosphomoribdic acid for 5 min, counterstained with aniline blue solution, and dehydrated multiple times with 95% ethanol. Image was obtained using an upright microscope (Nikon).

### Hematoxylin–eosin stain

Lung tissue slices were deparaffinized and rehydrated in xylene, stained with hematoxylin eosin, and differentiated using 1% ethanol hydrochloride, so that excessive staining solution bound in the nucleus and excess staining solution in the cytosol were removed. The image was obtained using an upright microscope (Nikon).

### Collagen gel contraction assay

Cells were suspended in serum-free media, then mixed with Collagen Type Ⅰ(Corning, 354236) and keep a final cell density about 1 × 105. Then, pipette the mixture into 24-well put it into 37 °C for 1 h. The edge of the gel was detached from the well walls with a pipette tip after the gel set, and 500 ul medium. After culture for 48 h, the area of gel contraction was measured using ImageJ software.

### Micro-CT imaging

Twenty-one days after bleomycin administration, *in vivo* micro-CT analysis of the whole lung was performed. Mice were lightly anesthetized with isoflurane and fixed in the supine position. Micro-CT images were acquired using a Bruker SkyScan 1276 micro-CT (Bruker, Kontich, Belgium). The scanning parameters were as follows: 60 kV X-ray tube voltage and 200 μA anode current; Cu filter of 0.5 mm, resulting in a total acquisition time of approximately 10 min. The reconstructed images were superposed by Insta-Recon software (Bruker microCT).

### Gene expression data

The analysis of CD147 gene expression was conducted using the GSE110147 data from the GEO database. The ggplot2 package (version 3.5.1) in R (version 4.4.2) was employed to draw the box plot, which intuitively presented the distribution characteristics of the data. The ggsignif package (version 0.6.4) was utilized to perform statistical tests on the data between groups. The "test" parameter was set as "wilcox.test" to execute the Wilcoxon test.

### Statistical analysis

All the experiments were conducted at least in triplicate. The data were presented as the means ± SEM or means ± SD. Data were analyzed with the use of an unpaired *t* test for comparisons between two conditions or ANOVA with the Tukey post-test to determine the differences among all groups. The data of *in vivo* experiments were analyzed with one-way ANOVA. The significance level was set at *p* < 0.05. Statistical analysis was performed using GraphPad Prism software (GraphPad Software).

## Ethics declarations

All animal procedures were approved and performed by the guidelines of the Institutional Animal Care and Use Committee at Henan Normal University (IACUC, SMKX-2118BS1018).

IPF lung tissues and control non-IPF lung tissue samples were recruited based on the ATS/ERS/JRS/ALAT Clinical Practice Guidelines at Henan Provincial Chest Hospital. The study was approved by the Henan Provincial Chest Hospital Medical Research Ethics Committee (No. 2019-05-07), and informed consent was obtained from all the patients before surgery. This research work was conducted in accordance with the relevant provisions on human subject research as stipulated in The Code of Ethics of the World Medical Association (*i*.*e*., the Declaration of Helsinki).

## Consent for publication

All authors have read and approved of its submission to this journal.

## Data availability

The data that support the findings of this study are publicly available at Gene Expression Omnibus (GEO) under the accession number GSE110147. All other data that support the findings of this study are available from the corresponding author upon request.

## Supporting information

This article contains supporting information.

## Conflict of interest

The authors declare that they have no conflicts of interest with the contents of this article.
