# Supplementary material for: Autophagy induced by glycosylated CD147 attenuates fibroblast activation and myofibroblast differentiation
Source: J Biol Chem. 2026 Jul 28;302(9):113374. doi: 10.1016/j.jbc.2026.113374 (PMC13524601; doi:10.1016/j.jbc.2026.113374)
Supplement: Supplementary file 1 [file mmc1.docx]

**Figure S1.** (**A**) Representative images of H&E (top) and Masson (bottom) stained paraffin sections of lung tissue from mice treated with BLM for 21 days, Scale bars, 50 µm. (**B**) Representative images of Fibronectin, COL1A1, and α-SMA in paraffin sections of lung tissue from mice treated with BLM for 21 days, Scale bars, 50 µm. (**C**) Immunohistochemical staining of CD147 in mice pulmonary fibrosis induced by BLM. (**D**) Representative results of the expression of fibrosis-related proteins and quantitative analysis of Fibronectin, COL1A1and α-SMA in the lung tissue of mice treated with BLM for 21 days. *****P<0.0001.*

**Figure S2.** (**A**) Western blot was conducted to assess the protein expression of MRC5 cells transfected with CD147 interference fragment and the corresponding quantitative analysis. (**B**) CCK-8 assay was used to measure cell viability in MRC-5 cells after knocking down CD147 expression. (**C**) CFU assay was employed to evaluate the representative image and quantitative analysis of colony-forming ability in MRC-5 cells after knocking down CD147 expression. (**D**) Western blot was conducted to assess the protein expression and perform quantitative analysis in MRC5 cells after overexpressing CD147. *ns: not significant, * p < 0.05 *** p < 0.001, **** p < 0.0001.*
